# The diffusion metrics of African swine fever in wild boar

**DOI:** 10.1038/s41598-023-42300-0

**Published:** 2023-09-13

**Authors:** Hartmut H. K. Lentz, Hannes Bergmann, Franz J. Conraths, Jana Schulz, Carola Sauter-Louis

**Affiliations:** Institute of Epidemiology, Friedrich-Loeffler-Institut, Federal Research Institute for Animal Health, 17493 Greifswald, Insel Riems, Germany

**Keywords:** Stochastic modelling, Computer modelling

## Abstract

To control African swine fever (ASF) efficiently, easily interpretable metrics of the outbreak dynamics are needed to plan and adapt the required measures. We found that the spread pattern of African Swine Fever cases in wild boar follows the mechanics of a diffusion process, at least in the early phase, for the cases that occurred in Germany. Following incursion into a previously unaffected area, infection disseminates locally within a naive and abundant wild boar population. Using real case data for Germany, we derive statistics about the time differences and distances between consecutive case reports. With the use of these statistics, we generate an ensemble of random walkers (continuous time random walks, CTRW) that resemble the properties of the observed outbreak pattern as one possible realization of all possible disease dissemination patterns. The trained random walker ensemble yields the diffusion constant, the affected area, and the outbreak velocity of early ASF spread in wild boar. These methods are easy to interpret, robust, and may be adapted for different regions. Therefore, diffusion metrics can be useful descriptors of early disease dynamics and help facilitate efficient control of African Swine Fever.

## Introduction

African swine fever virus (ASFV) causes an internationally spreading haemorrhagic pig disease with a massive socio-economic impact^1,2^. The current African swine fever (ASF) pandemic originated from disease incursion of genotype II ASFV in Georgia during 2007^3^. From there, ASF spread northwards into the Caucasus region, then further disseminated westwards into Europe, eastwards into Southeast Asia^2^, and even jumped across the Atlantic to threaten the Americas with outbreaks reported in the Dominican Republic and Haiti in 2021^4^. Since the start of the pandemic, an estimated quarter of the global domestic pig population has been decimated by the disease, causing food insecurity and economic losses on an unprecedented global scale^5–7^. Particularly during the early phases following new ASF incursion, well informed anticipation of disease spread is critical for controlling the disease efficiently.

As a consequence of the incursion into Georgia in 2007, ASF (genotype II) reached the territory of the European Union (EU) in 2014, when the first ASF cases were reported in wild boar in Lithuania and Poland^8–10^. Since then, and despite ongoing control efforts as well as intensive study of the disease dynamics, ASF has been moving predominantly in a western direction, affecting many more EU countries^11^. Among them was Germany, which reported its first ASF cases in 2020^12^. At that time, ASF had entered the country along a wide front on its eastern border, with several incursions detected in distinct areas, in which the disease initially continued to spread by forming relatively isolated spatial clusters^13^. Following the early detections of ASF in wild boar, the incursion sites were subsequently managed as local circumstances permitted^13^. Control measures included the implementation of zoning, disease surveillance, carcass searches and removal, strategic fencing and wild boar density reduction^12–14^.

In eastern and central Europe, wild boar seem to represent the predominant, disease-sustaining reservoir host in the current European ASF scenario. This is based on the spatial extent of cases in this pig type^15^, as well as their critical role in disease transmission through persistence of virus in the environment^2,16–18^. ASF is characterised by a case/fatality ratio of over $> 90$%. The carcasses of infected wild boar that succumbed to the disease may harbour infectious virus for weeks, if not months, thus contributing to the environmental contamination with ASFV^18,19^.

Unexpected occurrences of wild boar-ASF cases in locations that are a long distance away from the nearest previously affected area, such as suspected point incursions into the Czech Republic^20^, Belgium^21^, into the western part of Poland^18^, or into Northern Italy^4^, indicate that ASF can be relocated in association with human activities. However, typically ASF spreads in a gradual manner through infections and dissemination of disease in wild boar at a local scale. On the one hand, rare long-distance disease jumps are extremely hard to predict as they are presumably caused by human activity^3^. On the other hand, common short distance spread of ASF is mainly driven by wild boar biology, and describing the underlying dynamics or processes is crucial for efficiently implementing counter measures.

Whilst ASF outbreaks in domestic pigs appear to be manageable in most countries, the gradual disease spread in wild boar is very difficult to control and often persists^22–24^. Based on historic ASF case reports, average disease spread velocities of approximately 1 to 1.5 km per month have been estimated^11,25,26^. Control measures that efficiently manage ASF dissemination following new incursions require risk-based allocation of limited resources and rely on disease spread predictions that are locally applicable to the acute outbreak situation in the field.

Most epidemiological, mechanistic models for African swine fever in wild boar depend on a large number of parameters and assumptions (see^27^ for a comprehensive overview). These mechanistic models often need to be complex in order to describe ASF transmission dynamics in exposed wild boar populations that help evaluate alternative control strategies or examine risk factors and transmission parametrisation. Due to their complexity, it is often difficult to draw practical conclusions from these models. For assessing African swine fever control, simple and easily understandable metrics are needed, such as the following: Given a new occurrence of ASF, (1) What is the affected area when the disease spreads?, (2) How far does the epidemic reach from the index case over time?, and (3) What is the velocity of spread?

In order to answer these questions, we take a perspective that is different from most predictive models: What if we leave the unknown contact details of the underlying transmission process implicit, and focus on describing the observed spatiotemporal pattern of outbreak points with a suitable simple random process? A random process models the random evolution of a system over time, such as the dispersion of disease events. Assuming that a random process generates the time gaps and distances between consecutive cases of ASF occurrence, we could use the mathematical description of this process to compute all the desired metrics that are described above.

Even though ASF dynamics seem to be complex in general, disease dissemination appears to follow a remarkably simple pattern when considered on a local scale. As such, local outbreak areas appear to be growing over time with new cases constantly emerging inside and in the near vicinity of the affected area. By contrast, long-distance disease jumps are only observed as rare and extreme events, indicating a distinct process underlying the long distance spread events when compared to localised disease dissemination^11^. It would therefore be useful to generate robust metrics that help quantify the common dynamics of local ASF outbreaks in wild boar to assess and manage disease control efforts.

One of the simplest stochastic processes that could model the random dispersion of disease events over time and space is diffusion. If we are able to formulate a basic model that captures the fundamental disease spread dynamics as a random process, it would be possible to deduct informative metrics that characterise disease spread. We therefore follow this idea to describe the epidemic as a pure diffusion process.

Logically, an epidemic is not a pure diffusion process, which typically refers to molecules, but not individual animals or epidemiological units during disease outbreaks. Diffusion describes concentration changes of molecules and their distribution in space and time. The underlying molecular dispersal process that generates diffusion patterns is a random process known as Brownian motion^28–30^. Nevertheless, the parallels between the principles of molecular diffusion and epidemic disease dispersal on a larger spatial scale are clearly apparent. Similar to diffusion of molecules, epidemics describe individuals, their interactions, disease states, and resulting disease distribution patterns in space and time. In the context of ASF in wild boar, individual cases form a local outbreak cluster, which corresponds to the spatial disease pattern that emerges from an underlying random process. As such, epidemics can be modelled in an accurate way by resembling diffusion. This allows for the description of a suitable random process generating disease events, which can be interpreted mathematically in a relatively simple fashion. Once the logic of this random process is understood and calibrated for the data, diffusion of disease spread can be extrapolated spatially and over time.

For a purely diffusive process, a similar approach has been used on human mobility data^31^. In the context of ASF, a probabilistic model considering random walks by wild boar with infection dynamics has been proposed previously^32^. In contrast to that model, we consider the process that generates the disease pattern itself as a random walk.

Another disease model considers the diffusion around a primary case and includes a habitat-suitability component in this work^33^. The diffusion component in^33^, however, is not time dependent and therefore the model is not suitable for temporal predictions. A predictive model for ASF has been proposed in^34^. This approach is based on a compartment model and is therefore suitable for a prediction. Nevertheless, assumptions are necessary for several parameters considered in this model and a spatial component is not included.

Besides epidemiological compartment models, individual based models have also been used to estimate the transmission parameters of ASF, based on real outbreak data^35^. This model contains detailed data, and the movement and infection dynamics are considered explicitly. Contrasting the model proposed in the present paper, however, the agent-based model model in^35^ by design requires a larger number of parameter assumptions. Finally, the local wave front velocity of ASF has been modelled for Belgium in^36^.

All of the mentioned models provide good insights on the dynamics of ASF. However, there have been no models yet, that capture the physics behind ASF outbreaks. This would allow us to spatially describe disease spread dynamics over time, based on general principles for apparently similar processes in nature. Despite the fact that ASF is an infection process, it appears as a pure diffusion process on the map. For this reason, we fit a diffusion model to the disease data in order to measure the diffusion parameters of the ASF epidemic directly.

## Material and methods

### Data

**Figure 1:**
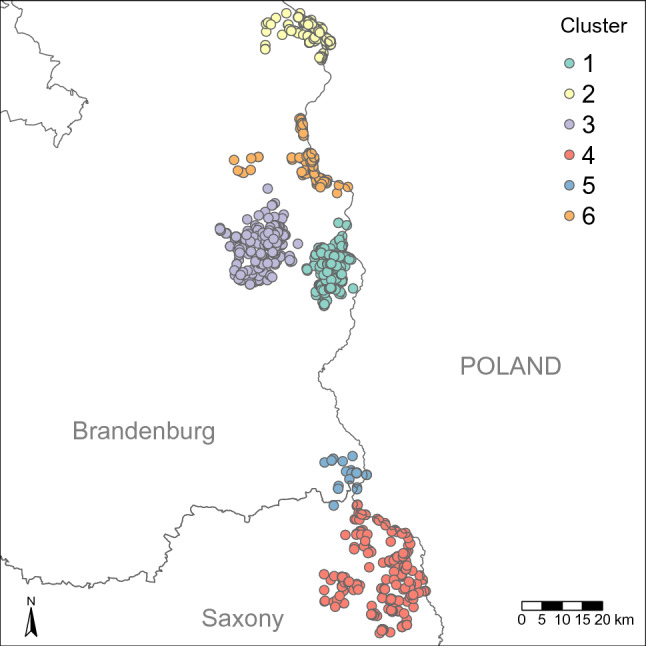
African swine fever case data and its separation into clusters. (Map was generated using R (version 4.2.8), packages maptools (version 1.1-7) and ggplot2 (version 3.4.2))^38^.

We use the official ASF case data for Germany covering all cases from 10 September 2020 to 9 July 2021 from the national animal disease database (Tierseuchennachrichtensystem)^37^. Due to the early phase of disease incursion into Germany, we were able to separate the data into clusters^13^, as shown in Fig. 1. The clusters of case locations were distinguished through euclidean distance-based, complete agglomerative clustering, implemented with the ‘hclust’ function from the R ‘stats’ package^38^. The resulting hierarchical tree was cut at a level that separated six case clusters, reflecting jurisdiction in the area. For simplicity in this paper, we will analyse cluster 1 as a representative cluster in detail. All other clusters show similar microscopic patterns (i.e. case distribution patterns, see Supplementary Information) and we compare all clusters briefly in the results section.

Each instance in the data set represents a *case*, i.e. time and coordinates of a detected ASF-positive wild boar. To distinguish between real data and data generated by our model, we use the term *event* for the random walk model instead of *case*. For clarity, we refer to a cluster of ASF-cases in wild boar as an *outbreak* (which should not be confused with occurrence of ASF in domestic pigs).

**Figure 2:**
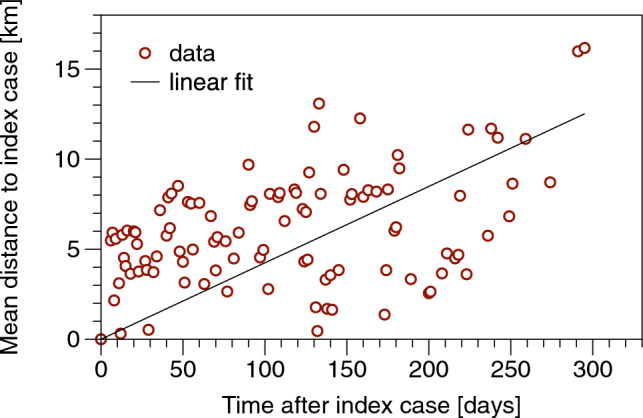
Distance to index case as a function of time. Every data point represents one case. Where multiple cases were detected at one day, the y-axis presents the mean distance. Fitted slope is $0.042 \; \mathrm {km/day}$.

In order to get a first simple estimate of the outbreak velocity, we calculate the distance from each case to the index case over time. This is shown in Fig. 2. Using a linear fit with an intercept that was fixed to zero, we obtain a velocity of $0.042 \; \mathrm {km/day}$.

As shown, this approach only makes the logical assumption that the relationship of time and distance to the index case is linear. Whilst this assumption appears to give a good initial estimate of the spread velocity, it does not capture all features of the disease spread dynamics.

### Brownian motion

In the present work we consider the outbreak data as points that are seemingly generated randomly in space. The only constraint is that new data points are generated in geographical and temporal closeness to existing points. We hereby establish the simple assumption that new data points are somehow related to existing data points. If we assume in the first place that every existing data point generates exactly one new data point at the next time step, the generating process would be Markovian. On closer consideration, however, this assumption is not valid, since waiting times occur between the cases, and thus secondary outbreaks can be later in the future.

Random walk processes that consider waiting times are called *continuous time random walks* (CTRW). Such a process works as follows: A random walker is initiated at time $t=0$ (time of the first case) at a location, say $(x_0, y_0) = (0, 0)$ (location of the first case). Then it waits for a random time $\tau _1$ and makes a jump of random length $l_1$ in a random direction. Thereafter, it waits for a random time $\tau _2$ and performs another jump of random length $l_{2}$ and so forth. We assume that jump lengths and waiting times are uncorrelated. Jump lengths are sampled from a distribution $\phi (l)$ and the waiting times from a distribution $\psi (\tau )$. In this work, the distributions of $\phi (l)$ and $\psi (\tau )$ are determined from the outbreak data. Hence, we generate synthetic outbreak data that is statistically equivalent to the observed data, by implementing the CTRW as follows:

- Start at the coordinates of the index case. Set these $(x_0, y_0) = (0, 0)$.
- Sample the waiting time from the waiting-time distribution $\psi (\tau )$ and generate a sequence of time points (event points) following the sampled waiting times.
- For each event point: sample a jump length from the jump-length distribution $\phi (l)$ and perform a step in a random direction.

The latest event determines the duration *T* of the random walk. We refer to one realisation of a complete CTRW as a *trajectory* *X*(*t*).

### Minimum spanning tree

To derive the jump length and waiting times between cases from the examined ASF case cluster and for a deeper understanding of the outbreak pattern, we consider the causal ordering of the cases in more detail. It is important to stress that the dataset itself does not contain any causal information between the cases.

Indeed, the measured data points represent an underlying – and unknown – infection tree that describes in detail which case has caused which other case(s).

Since the exact relationships in this infection tree data are unknown, we estimate causality in the following way (a similar idea was used in^39^):

1. Sort cases by time.
2. Generate a directed acyclic graph (DAG) $T=(V(t), E)$ with edge set $E = \emptyset$, where each node $v(t) \in V(t)$ is a case with time stamp *t*.
3. Connect nodes in *T* with directed edges from case *s* to case *t* as follows: whenever the target case *t* is after or at the same time as the source case *s*, draw a directed edge (*s*, *t*). Thus, the added edges $E \ne \emptyset$ in T comprise all possible causal connections between the cases.
4. Weigh all edges with the reciprocal geographical distance between the respective nodes/cases. (Vanishing distances are assigned a weight of zero.)
5. Finally, compute a *minimum spanning tree* on the now weighted DAG. For this, we used the Chu-Liu/Edmond Algorithm^40,41^ implemented in^42^.

This procedure orders the cases in a causal and geographically plausible manner. Using the minimum spanning tree, we obtain the distances between the cases and from those distances the jump length distribution. Subsequently, we also apply kernel density estimation to this distribution for smoothing. This allows sampling from a continuous distance distribution, as the distribution of observed jump lengths in the field data is discrete.

The empirical distribution of waiting times was directly derived from the outbreak data. We sort the cases by time and compute the differences between consecutive cases yielding the waiting time distribution.

For the waiting times, sampling is conducted from the empirical data and for jump distances the smoothed, kernel density estimations were sampled directly to generate the sequence of timepoints and jump lengths that implement the continuous time random walk to generate a random walk *trajectory* *X*(*t*).

### Time correction in random walk

It is important to emphasise that, in contrast to an epidemic process, a random walker trajectory can only be at one location at a time. By contrast, an epidemic can be at multiple locations at the same time, that is, an epidemic can branch out into multiple locations simultaneously. To resolve this problem, we correct the time available to the random walker for moving around. If multiple cases are observed in one day, but a random walker can only generate one event per day, then the allowed time for the random walk has to be increased to allow the random walker to generate the required number of events one after the other. We thus use the following idea:

Let the total random walk have a maximum duration of *T*. In the easiest situation, exactly one case occurs per day. Now consider the situation where an average of *M* cases occur per day. Then the random walker must have the ability to generate these cases/events without spending time. We call *M* the *multiplicity* of the process. As an example, if we have $M=3$ cases per day and the maximum duration of the whole random walk is $T=100$ days, then the random walker is given $MT=300$ available days for generating the required 300 events in total. Finally, in order to return to the original time scale, we rescale the new maximum duration (300 days) back to the initial value (100 days). For simplicity, we refer to the time steps taken by the model also as ’days’, although the exact duration of each simulated realisation may vary. The duration of a complete realisation depends on the cumulative waiting times randomly drawn from the waiting time distribution. Multiplicity ensures that the required number of events is generated, by giving the model the number of time steps needed to do so.

### Diffusion coefficient, expected radius, and velocity

The Brownian motion described above is a single realisation of a microscopic random process (movement of molecules). Averaging over a large number of random walks yields the macroscopic properties of the process (emerging pattern). Since every random walker can walk in a different direction, the expected location is $\langle X(t)\rangle =(x_0, y_0)= 0$ for all times *t* (the brackets $\langle \cdot \rangle$ refer to the average over all random walkers).

For large times *t* a single random walker is expected to be located at a great distance from the origin. Therefore, using variance of random walker displacement as a measure of dispersion, the *mean squared displacement* (MSD) $\langle X(t) ^2 \rangle$ increases with time. The detailed form of the MSD has to be determined empirically. In case the MSD follows a linear relation, i.e. $\langle X(t) ^2 \rangle \sim t$, the corresponding macroscopic process is called *normal diffusion*. In that case

1 $$\begin{aligned} \langle X(t) ^2 \rangle = 4 D t \end{aligned}$$

and the constant *D* is the *diffusion coefficient*. Equation (1) represents the variance of the random walkers’ positions after time *t*.

The square root of the MSD is the expected radius, the mean distance by which all random walkers are expected to be dispersed from the origin case after time *t*, i.e.

2 $$\begin{aligned} r(t) = \sqrt{\langle X(t) ^2 \rangle } = \sqrt{4Dt}. \end{aligned}$$

We identify this quantity with the radius of the affected area or the distance between the index case and the wave front.

Finally, the velocity of the wave front *v*(*t*) can be defined as the change of the radius with respect to time, thus

3 $$\begin{aligned} v(t) = \frac{dr(t)}{dt} = \sqrt{ \frac{D}{t}}. \end{aligned}$$

Note that *r*(*t*) and *v*(*t*) are not linear.

Our implementation of the mentioned methods is available online^43^.

## Results

**Figure 3:**
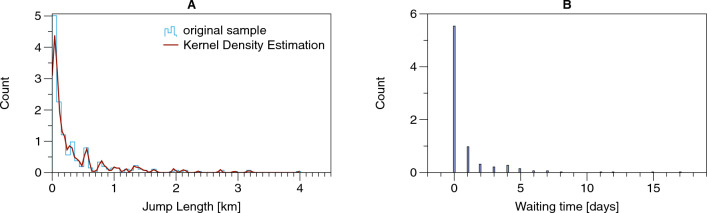
Jump length distribution (**A**) and waiting time distribution (**B**) derived from the outbreak data of Cluster 1. (The jump length distribution is for cases ordered using the minimum spanning tree). The blue histogram in (**A**) shows the observed counts of jump length and the red line a smoothed kernel density estimate distribution of this data for sampling in the diffusion model.

To bring all cases into a plausible order, we first sort the outbreak data by detection time and compute the minimum spanning tree. This tree provides us with the distribution of shortest jump lengths. We then generate the waiting time distribution directly from the outbreak data. Both distributions are shown in Fig. 3.

**Figure 4:**
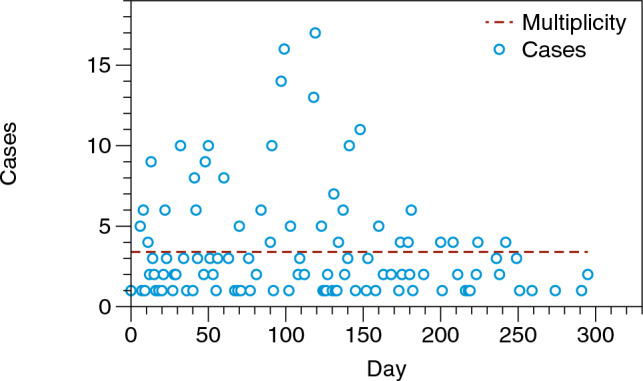
Number of cases per day of cluster 1. The multiplicity is 3.4.

A random walker cannot be in multiple locations at the same time. On the contrary, for epidemic processes multiple cases can occur simultaneously. This is exemplified for Cluster 1 in Fig. 4.

On average 3.4 cases occur per day over 300 days in total, i.e. the multiplicity of the process is $M=3.4$. Therefore, we multiply the available time for the random walker by *M*. This yields 1020 time steps which are afterwards rescaled to 300 days.

**Figure 5:**
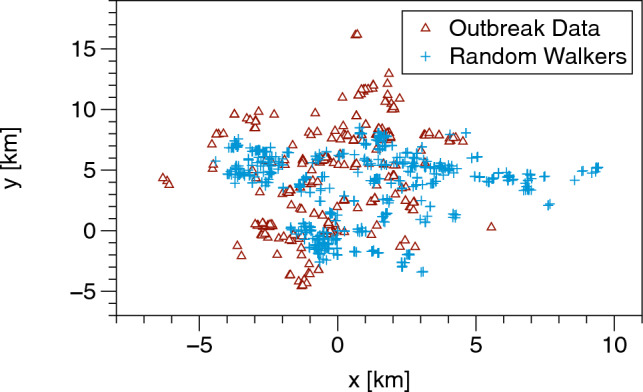
Real outbreak data vs. one realization of a random walk for Cluster 1. The index case is set to coordinates (0, 0). Outbreak data from 300 days, random walk with multiplicity 3.4 resulting in 1020 steps that represent 300 days.

Using the distributions from Fig. 3 and the multiplicity *M* we generate an ensemble of 10,000 random walkers to guarantee statistical stability. In order to get an impression of the microscopic properties of the random walks (localisation of individual events), we show one realization in Fig. 5.

This realization appears to show considerable structural similarity to the observed outbreak data, in the sense that both sets of points appear to be sampled from a similar distribution. Note that a random walk is an isotropic process, which means that all directions are equally likely. It is therefore plausible that the outbreak case data and the synthetic event points can be spread out in different directions as long as they have a similar structure.

**Figure 6:**
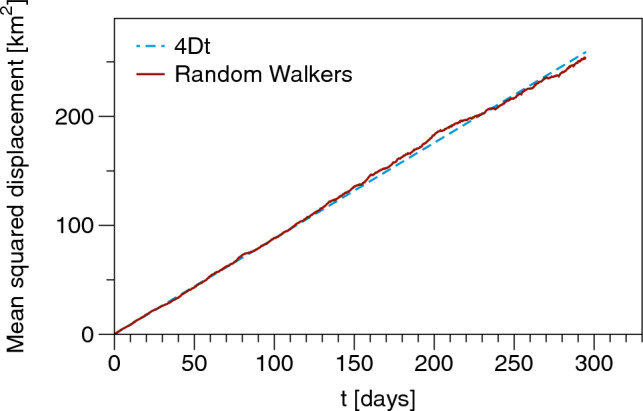
Mean squared displacement for an ensemble of 10,000 random walkers (red line). The resulting diffusion constant *D* follows from a linear fit (blue dashed line) which gives $D = (0.22 \pm 0.01) \; \textrm{km}^{2} / \textrm{day}$.

We now study the macroscopic (diffusion) properties of the random walker ensemble. Figure 6 shows the mean squared displacement (MSD) over an ensemble of 10,000 random walkers. The MSD follows a linear form indicating that the measured distributions result in a normal diffusion process. Using a linear fit, we obtain a diffusion coefficient of $D = (0.22 \pm 0.01) \; \textrm{km}^{2} / \textrm{day}$. This value is a median over all realizations and the error is the inter-quartile range.

**Figure 7:**
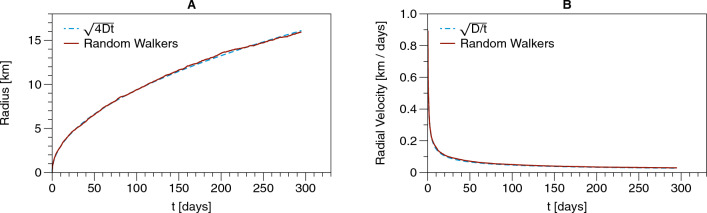
(**A**) Radius of the area where all random walkers are likely to be contained after time *t*. (**B**) Radial velocity of the area growth. Red lines are mean values over the random walker ensemble, blue dashed lines are analytical, using the diffusion constant.

The radius of the affected area follows the square root shaped relation shown in Fig. 7A. After a steep increase in the early phase of the epidemic, the radius grows over time, but the front velocity decreases. Note that these features of disease spread dynamics, such as the slowing down of the wave front, cannot be captured by the simple linear approach used in Fig. 2. The wave front velocity over time is shown in Fig. 7B. The latter shows a quasi-constant behavior in the time scale of interest, i.e. roughly 0.04 km/day measured 150 days after the first case.

### Comparison between the clusters

**Figure 8:**
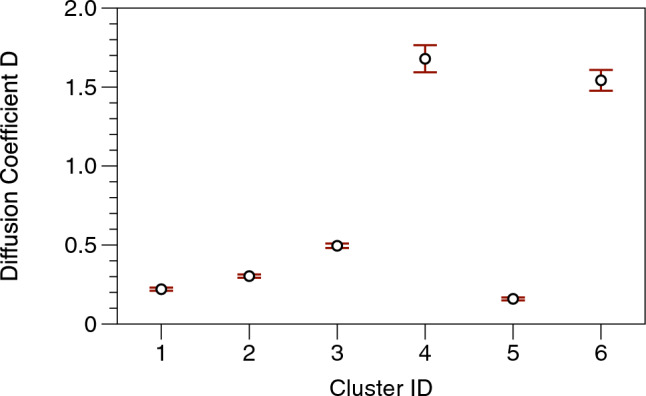
Diffusion constants for all clusters. Error bars show the inter-quartile distance. Each data point is for 10,000 simulations.

So far, we have only studied one selected cluster. In Fig. 8 we show the diffusion coefficients for all clusters. Each value is a median over 10,000 simulations. The error bars represent the inter quartile ranges.

The figure demonstrates that even if there are certain differences in the clusters, their diffusion coefficients show remarkable similarity. Most diffusion constants lie in a band between 0.2 and 0.5 $\textrm{km}^{2} /\textrm{day}$.

We provide a detailed description of the diffusion metrics for all clusters in the Supplementary Information.

## Discussion

In the present study we have considered the outbreak propagation of African swine fever as a diffusion process. Instead of making assumptions on wild boar movements, we focussed on the process that generates the outbreak data directly. Although this is an abstract concept, it allows us to measure the physical properties of the observed outbreak pattern. This concept is in contrast to previous work by Dellicour and colleagues, who measured the outbreak wavefront velocity by tracking the occurrence of new cases at the edge of an ASF cluster in time^36^. Thereby, the observed outbreak pattern and its edge velocity through the given landscape could be characterised, but alternative realisations of these spread events were omitted.

Assuming that the outbreak propagation follows a random walk appears drastic, since in contrast to a random walk, new cases can appear at multiple locations at the same time. This could be modelled by a branching process, where the random walker can reproduce itself and new events would occur simultaneously, as observed in real epidemics. Alternatively, more time can be allocated for a random walk to simulate comparable event numbers sequentially, whilst the random walker is only in one location at any one time. We implemented both possible approaches side by side when developing the model by also running Monte-Carlo simulations that included branching. We found that the resulting cluster realisations for branching were indistinguishable from sequential random walks (corrected by multiplicity) regarding their mean square displacement (not shown). This indicated that a branching process is not required for epidemic-like case dispersal patterns that generate multiple events simultaneously and that both approaches (simultaneous and sequential random dispersal) lead to the same outcome, given the condition that random walkers are not restricted in their possible location.

It could also be considered that infected animals that die block further disease spread (back) into the affected area, since susceptible individuals are removed from the population. This assumption would restrict the possible location of the random walker in our model. This situation could be implemented by using an annihilation process, where new random walkers can be restricted in their movement by avoiding closeness to already removed (’died’) random walkers. However, in the context of ASF outbreaks during the early phase, infected animals can freely move and also return to already infected areas. Therefore and in the context of the localised early outbreak phase, no restriction to the possible location of infected wild boar applies. In other words, the disease cannot be pushed out of already infected areas. Consequently, we do not consider restrictive random walks through annihilation in our model.

Although our results provide simple metrics for the propagation of ASF, the computation of these metrics is not trivial in general. On the one hand, estimating the wave front velocity using the simple linear distance to the index case has turned out to give a value remarkably similar to that of our model, thus confirming its plausibility. On the other hand, this simple approximation only provides an average estimate of spread velocity across the entire outbreak period, but does not capture detailed changes of radial velocity, such as the slowing of the wave front with time. Through considering the causal infection tree structure of the outbreak data, this level of detail is predicted by our model and may contribute valuable insights for the implementation of ASF control measures in wild boar. One critical realisation implied by the diffusion model is the initially quick dissemination of detected cases following discovery of a new ASF incursion in a previously disease-free area and the subsequent slowing of disease spread later in the epidemic (Fig. 7). This observation suggests, that upon first detection the disease may have already spread much further than perhaps anticipated, thus requiring adequately spaced counter measures to prevent falling behind and maintaining a chance of controlling a new outbreak as early as possible. Likewise, the anticipated slowing of disease spread at a later stage as an inherent feature of the dynamic process has to be evaluated against the actual effects of control measures.

For the random walk model, besides the needed Monte-Carlo-simulations, finding the distributions for waiting times and jump lengths requires manual adjustments. These could be optimised using a hyper-parameter-tuning scheme. For the obtained diffusion model results to be most representative of the observed situation in the field, constraints in the landscape (rivers, roads, fences, etc.) that potentially influence disease spread dynamics should be minimal. If more of these constraints are judged to be present in an examined outbreak cluster, more manual adjustment of the modelled process would be required to capture such constraints. Similarly, constraints not represented in the modelled diffusion process could originate from biased availability of the considered data (e.g. through limited jurisdiction or knowledge of the underlying surveillance effort) or animal population structures, rather than being caused by truly occurring geomorphological factors present in the landscape. Whilst we do not see this as a limitation of the modelled diffusion process itself, it is important to be aware of such constraints when interpreting the results. It would be an interesting and useful expansion of the model to explore factors in the landscape, the data or animal biology that may influence the observed ASF spread patterns in the field, including directionality.

If directional disease propagation in relation to constraints is of interest, in particular, this could be done either on a case by case basis, or by examining the directionality that emerges for each simulated iteration of the entire cluster. It should be possible to measure the turning angle of direction between subsequent cases or between congruencies of outbreak pattern iterations. Such an expansion of the model would predict the disease wave front expansion into a certain direction, as implied by the statistical characteristics of the examined outbreak cluster.

Regarding the case waiting time data, clear limitations for the underlying dates entered into the model have to be considered. Here, the laboratory diagnostic conformation dates were available for all cases and consequently interpreted as the time when the observed ASF epidemic process generated a case. This is only an approximation of reality. In the field, wild boar succumb to ASFV infection, representing the time of death. Time elapses until the carcass is found and a sample is taken, the post-mortem interval, then additional time passes until the laboratory confirms detection of ASFV in the sample. Variability in post-mortem intervals and time to diagnostic confirmation biases the temporal case data. Nevertheless, the random walk approach still appears useful, since the temporal effects of ASF management within each disease cluster with regards to carcass searches and diagnostic procedures are reasonably expected to be consistent. In future, it would be intriguing to address this limitation by applying and accounting for the minimal post-mortem-interval in each case, thus aligning the temporal aspect of the model closer with disease dynamics in the field^44^.

As we have demonstrated in Fig. 8, the properties of some of the clusters are remarkably similar. This seems to be reasonable, as the counter measures implemented overall are similar in all of those clusters. Nevertheless, Clusters 4 and 6 show higher diffusion coefficients. In the case of Cluster 4, this could be due to the fact that the time needed for fences to be erected was longer than in other cluster areas. Moreover, the first cases occurred along an extended area of the border without any expansion for the first 80 days. For Cluster 6, a higher diffusion coefficient could have been caused by the fact that the disease occurred in an urban area, which did not allow for implementation of the same control measures as in the other clusters. Moreover, the different diffusion coefficients might be caused through cases occurring along an extended area at the German-Polish border, thus showing a high degree of constraint in these clusters (see Fig. 1, and Supplementary Information for more details) It is important to stress the fact that this constraint is caused primarily by the data availability and not by the underlying process. That is, we would expect to get a more consistent picture here, if Polish data would have been included in the analysis.

A key value of the methods presented herein, is the ability to randomly generate synthetic outbreak patterns from the causal ordering of an infection tree that logically resembles the observed cluster-specific case pattern in the field. This simulation could be useful for many applications, such as the assessment of mechanistic disease transmission factors or for testing of disease control measures. Here, we applied the outbreak informed simulations to investigate spread velocity of ASF in wild boar. Consequently, we state that the observed patterns follow a general mechanism, at least for the examined date set, representing a particular area in Germany. In conclusion, it seems possible to derive a general diffusion law for this kind of setting, which might be helpful for disease control.

### Supplementary Information

Supplementary Information.

## Data Availability

The datasets used and analyzed during the current study are available from the corresponding author on reasonable request.

## Code availability

The code is available online on https://github.com/hartmutlentz/RandomWalker2D.
