# Supplementary material for: The diffusion metrics of African swine fever in wild boar
Source: Sci Rep. 2023 Sep 13;13:15110. doi: 10.1038/s41598-023-42300-0 (PMC10499946; doi:10.1038/s41598-023-42300-0)
Supplement: Supplementary file 1 — Supplementary Information. [file 41598_2023_42300_MOESM1_ESM.pdf]

Supplementary Information – The Diffusion Metrics of African Swine Fever in Wild Boar

Hartmut H. K. Lentz, Hannes Bergmann, Franz J. Conraths, Jana Schulz, Carola Sauter-Louis

Friedrich-Loeffler-Institut, Federal Research Institute for Animal Health, Institute of Epidemiology, 17493 Greifswald - Insel Riems

1 Supplementary Information

1.1 Wave front velocities

We compare the estimated velocities found by simple linear regression as done in the main text. The result is shown in Figure S1. As expected, the velocities are related to the diffusion coefficients for the considered clusters, i.e. they show similar values, except Clusters 4 and 6.

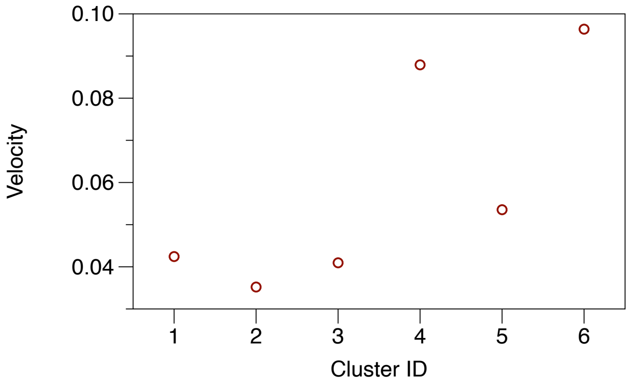

Figure S1: *Velocities for all clusters estimated using linear regression of the distances to the index case.*

1.2 Mean squared displacements of Clusters 2–6

1.2.1 Cluster 2

Figure S2 shows a realization of a random walk trained on the outbreak data. The multiplicity of the process is 4.1.

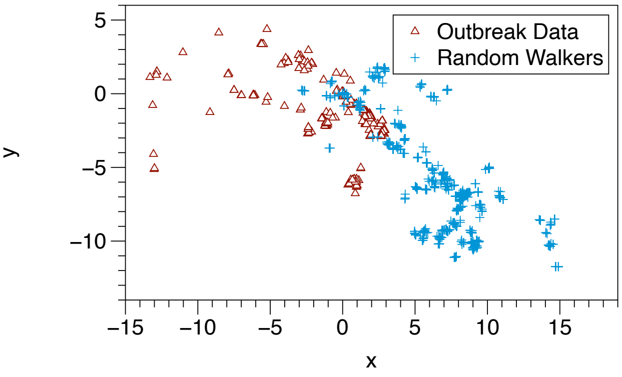

Figure S2: *Real outbreak data vs. one realization of a random walk for Cluster 2. The index case is set to coordinates (0, 0). Outbreak data from 265 days, random walk with multiplicity 4.1 resulting in 1087 steps.*

We show the mean squared displacement in Figure S3. The diffusion coefficient is $D = (0.30 \pm 0.01)\ km^2/day$.

1.2.2 Cluster 3

Figure S4 shows a realization of a random walk trained on the outbreak data. The multiplicity of the process is 3.6.

We show the mean squared displacement in Figure S5. The diffusion coefficient is $D = (0.50 \pm 0.01)\ km^2/day$.

1.2.3 Cluster 4

Figure S6 shows a realization of a random walk trained on the outbreak data. The multiplicity of the process is 4.7.

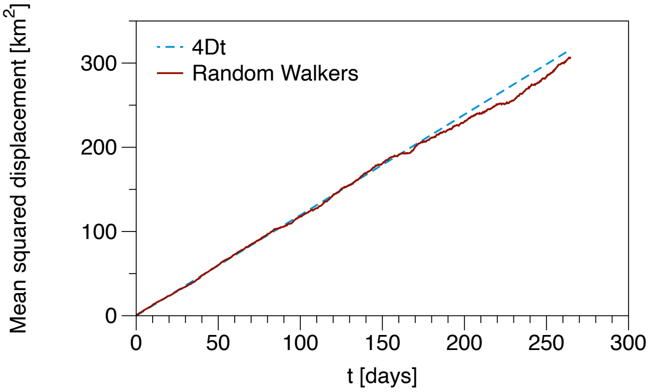

Figure S3: *Mean squared displacement for Cluster 2. Mean over 10,000 random walkers (red line). The resulting diffusion constant is* $D = (0.30 \pm 0.01)\ km^2/day$.

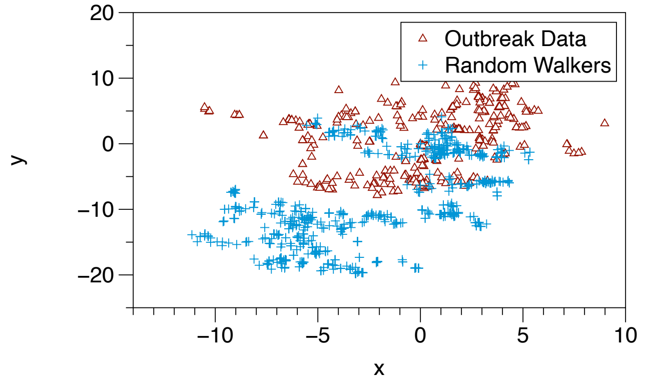

Figure S4: *Real outbreak data vs. one realization of a random walk for Cluster 3. The index case is set to coordinates (0, 0). Outbreak data from 245 days, random walk with multiplicity 3.6 resulting in 882 steps.*

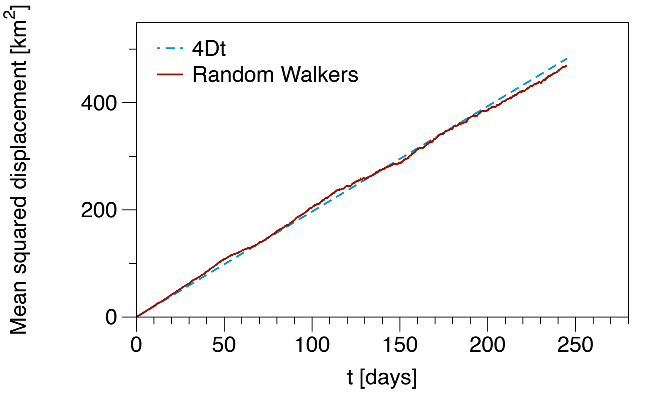

Figure S5: *Mean squared displacement for Cluster 3. Mean over 10,000 random walkers (red line). The resulting diffusion constant is* $D = (0.50 \pm 0.01)\ km^2/day$.

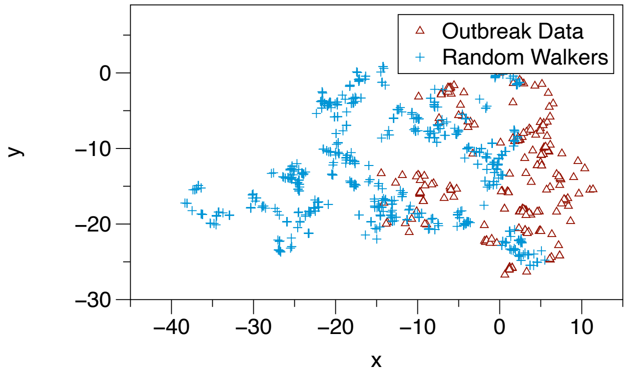

Figure S6: *Real outbreak data vs. one realization of a random walk for Cluster 4. The index case is set to coordinates (0, 0). Outbreak data from 249 days, random walk with multiplicity 4.7 resulting in 1170 steps.*

We show the mean squared displacement in Figure S7. The diffusion coefficient is $D = (1.69 \pm 0.09)\ km^2/day$.

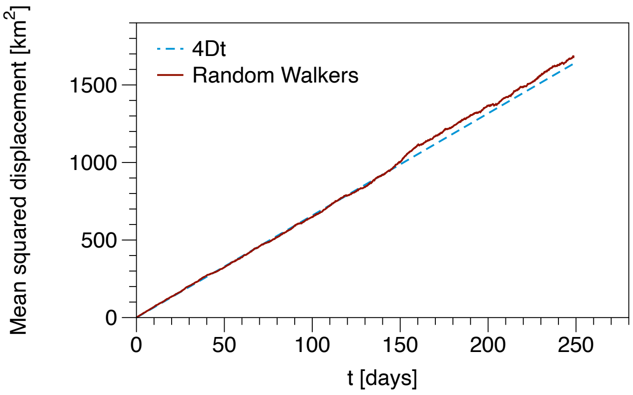

Figure S7: *Mean squared displacement for Cluster 4. Mean over 10,000 random walkers (red line). The resulting diffusion constant is* $D = (1.69 \pm 0.09)\ km^2/day$.

1.2.4 Cluster 5

Figure S8 shows a realization of a random walk trained on the outbreak data. The multiplicity of the process is 3.0.

We show the mean squared displacement in Figure S9. The diffusion coefficient is $D = (0.16 \pm 0.01)\ km^2/day$.

1.2.5 Cluster 6

Figure S8 shows a realization of a random walk trained on the outbreak data. The multiplicity of the process is 6.2.

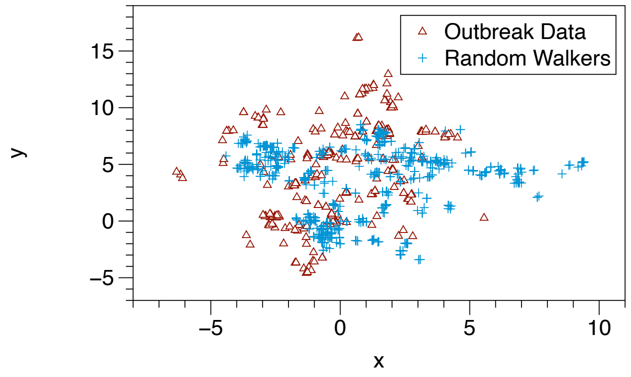

Figure S8: *Real outbreak data vs. one realization of a random walk for Cluster 5. The index case is set to coordinates (0, 0). Outbreak data from 121 days, random walk with multiplicity 3.0 resulting in 363 steps.*

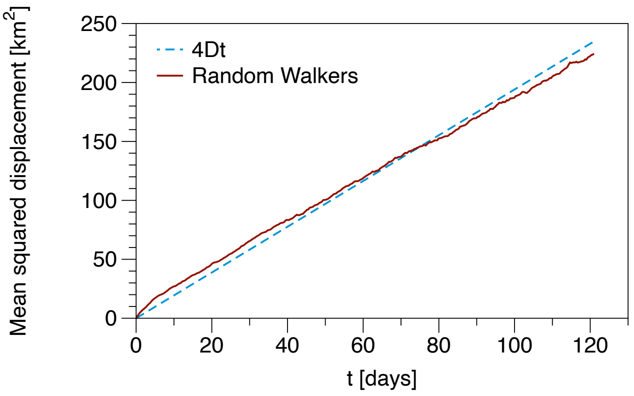

Figure S9: *Mean squared displacement for Cluster 5. Mean over 10,000 random walkers (red line). The resulting diffusion constant is* $D = (0.16 \pm 0.01)\ km^2/day$.

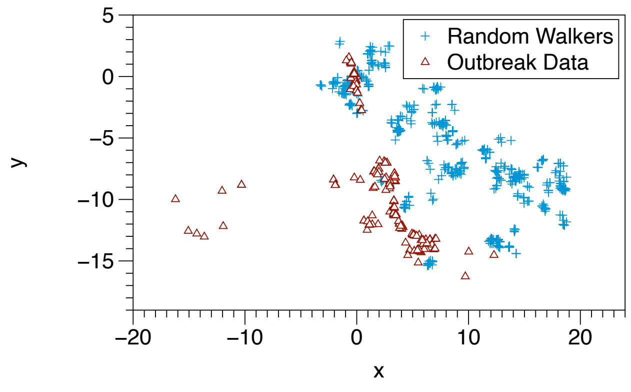

Figure S10: *Real outbreak data vs. one realization of a random walk for Cluster 6. The index case is set to coordinates (0, 0). Outbreak data from 127 days, random walk with multiplicity 6.2 resulting in 787 steps.*

We show the mean squared displacement in Figure S11. The diffusion coefficient is $D = (1.54 \pm 0.07)\ km^2/day$.

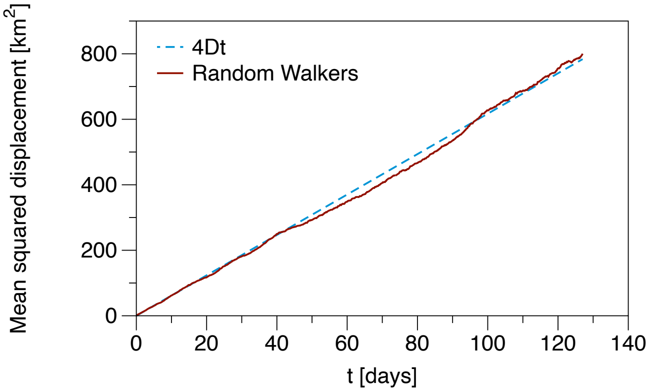

Figure S11: *Mean squared displacement for Cluster 6. Mean over 10,000 random walkers (red line). The resulting diffusion constant is* $D = (1.54 \pm 0.07)\ km^2/day$.

1.3 Summary and discussion of Clusters 2–6

The Clusters 2, 3, and 5 show a behavior similar to Cluster 1 in the main text. Their multiplicities are relatively low and most realizations of random walks appear very similar to the real outbreak data.

Clusters 4 and 6 show remarkable differences between synthetic and real outbreak data, as shown in Figures S6 and S10. The figures demonstrate that the generated data points cover a larger area when compared to the more compact outbreak data. This is caused by the fact that both clusters – and Cluster 6 in particular – are strongly geographically constrained by data being restricted to within the German country borders. In particularly, Cluster 6 is located along the river Oder. This implies that a large proportion of cases on the polish side is missing in the cluster.

Since the random walk model does not take into account such geographical constraints, the random walkers move in all directions ignoring the constraints. As a consequence, they cover a much larger area (take the eastern regions in Figure S10 as an example) and thus the diffusion constant is magnified. Cluster 4 shows a similar behavior, even if to a weaker extent (Figure S6).

An additional bias in Cluster 6 is the high multiplicity of 6.2. This is the highest value among all clusters and it causes a strong bias in the random walk model, since the random walker has to cover

more than 6 events occurring in the data each day. However, the random walk assumption only holds for multiplicities close to 1. Moreover, Cluster 6 is located in an urban area and consequently this restriction did not allow for the same control measures as in the other clusters.
